# RETRACTED: Al-Hussain et al. Application of New Sodium Vinyl Sulfonate–co-2-Acrylamido-2-me[thylpropane Sulfonic Acid Sodium Salt-Magnetite Cryogel Nanocomposites for Fast Methylene Blue Removal from Industrial Waste Water. *Nanomaterials* 2018, *8*, 878

**DOI:** 10.3390/nano16130822

**Published:** 2026-07-03

**Authors:** Sami A. Al-Hussain, Ayman M. Atta, Hamad A. Al-Lohedan, Abdelrahman O. Ezzat, Ahmed M. Tawfeek

**Affiliations:** 1 Department of Chemistry, Faculty of Science, Al Imam Mohammad Ibn Saud Islamic University (IMSIU), Riyadh 11451, Saudi Arabia; sahussain@imamu.edu.sa; 2 Surfactants Research Chair, Chemistry Department, College of Science, King Saud University, Riyadh 11451, Saudi Arabia; hlohedan@ksu.edu.sa (H.A.A.-L.); ao_ezzat@yahoo.com (A.O.E.); atawfik@ksu.edu.sa (A.M.T.)

The journal retracts the article titled “Application of New Sodium Vinyl Sulfonate–co-2-Acrylamido-2-me[thylpropane Sulfonic Acid Sodium Salt-Magnetite Cryogel Nanocomposites for Fast Methylene Blue Removal from Industrial Waste Water” [1], cited above.

Following publication, concerns were brought to the attention of the publisher concerning the integrity of several figures presented in this article [1].

According to the journal’s standard procedures, the Editorial Office and Editorial Board conducted an investigation that identified indications of inappropriate editing in Figures 2, 3 and 5. While the authors collaborated during the investigation and provided supporting materials, the Editorial Board concluded that the concerns could not be resolved satisfactorily based on the available information.

Consequently, the Editorial Board has lost confidence in the reliability of the reported findings and has decided to retract this publication [1], as per MDPI’s retraction policy.

This retraction was approved by the Editor-in-Chief of the journal *Nanomaterials*.

The authors have been informed of this retraction but did not provide a comment on this decision.

## References

[1] Al-Hussain S.A., Atta A.M., Al-Lohedan H.A., Ezzat A.O., Tawfeek A.M. (2018). RETRACTED: Application of New Sodium Vinyl Sulfonate–co-2-Acrylamido-2-me[thylpropane Sulfonic Acid Sodium Salt-Magnetite Cryogel Nanocomposites for Fast Methylene Blue Removal from Industrial Waste Water. Nanomaterials.

